# Preparation and *in vivo* characterization of ^51^MnCl_2_ as PET tracer of Ca^2+^ channel-mediated transport

**DOI:** 10.1038/s41598-017-03202-0

**Published:** 2017-06-08

**Authors:** Stephen A. Graves, Reinier Hernandez, Hector F. Valdovinos, Paul A. Ellison, Jonathan W. Engle, Todd E. Barnhart, Weibo Cai, Robert J. Nickles

**Affiliations:** 1 Department of Medical Physics, University of Wisconsin, 1111 Highland Ave., Madison, 53705 WI USA; 2 Carbone Cancer Center, University of Wisconsin, 1111 Highland Ave., Madison, 53705 WI USA; 3 Department of Radiology, University of Wisconsin, 1111 Highland Ave., Madison, 53705 WI USA

## Abstract

Manganese has long been employed as a T_1_-shortening agent in magnetic resonance imaging (MRI) applications, but these techniques are limited by the biotoxicity of bulk-manganese. Positron emission tomography (PET) offers superior contrast sensitivity compared with MRI, and recent preclinical PET studies employing ^52g^Mn (t_1/2_: 5.6 d, β^+^: 29%) show promise for a variety of applications including cell tracking, neural tract tracing, immunoPET, and functional β-cell mass quantification. The half-life and confounding gamma emissions of ^52g^Mn are prohibitive to clinical translation, but the short-lived ^51^Mn (t_1/2_: 46 min, β^+^: 97%) represents a viable alternative. This work develops methods to produce ^51^Mn on low-energy medical cyclotrons, characterizes the *in vivo* behavior of ^51^MnCl_2_ in mice, and performs preliminary human dosimetry predictions. ^51^Mn was produced by proton irradiation of electrodeposited isotopically-enriched ^54^Fe targets. Radiochemically isolated ^51^MnCl_2_ was intravenously administered to ICR mice which were scanned by dynamic and static PET, followed by *ex vivo* gamma counting. Rapid blood clearance was observed with stable uptake in the pancreas, kidneys, liver, heart, and salivary gland. Dosimetry calculations predict that 370 MBq of ^51^Mn in an adult human male would yield an effective dose equivalent of approximately 13.5 mSv, roughly equivalent to a clinical [^18^F]-FDG procedure.

## Introduction

Positron emitting isotopes of manganese (^52g^Mn, ^52m^Mn, and ^51^Mn) show promise for a variety of positron emission tomography (PET) medical applications. Divalent manganese cations have been shown to behave similarly to calcium biologically, allowing for free diffusion through voltage-dependent calcium channels (VDCCs)^1, 2^. This characteristic of manganese has allowed for investigations into neural tract tracing and functional β-cell mass determination^3–8^. Manganese is rapidly chelated by DOTA (1,4,7,10-tetraazacyclododecane- 1,4,7,10-tetraacetic acid) and appears stably conjugated for at least 5 days post-injection in murine studies^9^, which has enabled previous immunoPET studies and may allow for future bioconjugate applications^9^. Furthermore, macroscopic quantities of stable manganese may be employed as a T1-shortening agent in manganese-enhanced magnetic resonance imaging (MEMRI)^10^. With the recent advances in PET/MRI scanner technology^11^, radio-manganese may enable future dual modal imaging techniques.

^52g^Mn (t_1/2_: 5.591 d, β^+^: 29.4%, Eβ_ave_: 0.24 MeV) can be produced in sufficient quantities (~500 MBq/h with 60 µA of 16 MeV protons) and radionuclidic purity (>99.5%) on a low energy cyclotron by irradiation of natural chromium metal^12^. Because of this accessibility, ^52g^Mn has been used extensively in preclinical research in recent years^13, 14^. The radioactive half-life of ^52g^Mn is conducive to national or international transport, and its soft positron energy offers superb image resolution, comparable to ^18^F. Unfortunately, several prominent high energy gamma emissions (744 keV, 936 keV, and 1434 keV) limit clinical relevance. Likewise, ^52m^Mn (t_1/2_: 21 min, β^+^: 96.6%, Eβ_ave_: 1.17 MeV) has a high-energy gamma emission (1434 keV) preventing clinical translation.

^51^Mn (t_1/2_: 46.2 min, β^+^: 97.1%, Eβ_ave_: 0.96 MeV) on the other hand has no prominent (>1%) gamma emissions and a half-life that is suitable for clinical studies. One additional consideration however is the dose contributed by the daughter radioactive decay of ^51^Mn, ^51^Cr (t_1/2_: 27.7 d) which emits a 320 keV gamma ray in 10% of decays. Although the clinical use of ^51^Mn appears promising, particularly for pancreatic β cell imaging, there has been little previous work done on producing ^51^Mn on low-energy medical cyclotrons. Daube *et al*., produced ^51^Mn by the ^50^Cr(d,n) reaction^15^ for use as a myocardial perfusion tracer, using ^50^Cr_2_O_3_ powder as the irradiation target material. Likewise, Klein *et al*. produced ^51^Mn by ^50^Cr(d,n) but with a ^50^Cr metal powder target^16, 17^. Manufacturing a robust target from enriched ^50^Cr_2_O_3_ powder or ^50^Cr-metal powder is challenging however, as neither Daube *et al*., nor Klein *et al*., were able to exceed 4 µA of irradiation current^18^. Lawrence *et al*. were successful in producing a thin (2.8 mg/cm^2^) ^50^Cr-metal target by electrodeposition on Au^19^. These targets appear quite robust, withstanding up to 65 µA of 14 MeV deuterons, however methods of electrodeposition and chromium recovery were not detailed. Radionuclidically pure ^51^Mn may also be obtained through the ^54^Fe(p,α) reaction pathway. To our knowledge, this route has not been previously investigated for the production of clinically relevant quantities of ^51^Mn. The aims of this work were to (A) develop and characterize ^51^Mn production methods by ^54^Fe(p,α), to (B) characterize the *in vivo* behavior of ^51^MnCl_2_ in healthy mice, and to (C) evaluate the dosimetric feasibility of clinical ^51^Mn studies.

## Materials and Methods

### Materials and Nomenclature

All reagents were obtained from commercial vendors and were used as received unless otherwise stated. Aqueous solutions were constituted in >18 MΩ/cm H_2_O. Tissue uptake of radioactivity is specified in standard uptake values (SUV), defined as the product of the percent of injected dose per gram of tissue (%ID/g * 100) and the body weight (g) of the subject. Unless otherwise stated, all values are specified as mean ± standard deviation (SD).

### ^54^Fe Target Fabrication and Irradiation

Targets were prepared by electrolytic deposition of isotopically enriched ^54^Fe metal (<100 mg) on Ag disc substrates (0.5 mm thick, 19 mm diameter), as previously described^20–22^. Briefly, ^54^Fe-enriched metal (99.93%, Isoflex USA, San Francisco, CA) was dissolved in HCl (6 M, 2–5 mL). To this solution 100 µl of 30% H_2_O_2_ was added to promote the Fe(III) oxidation state. This solution was taken to near dryness (<1 mL), before adding 15 mL of saturated ammonium oxalate solution (stock solution stored with ~1 g Chelex® 100 resin to minimize trace metal impurities). Approximately 100 mg of L-ascorbic acid was added to this solution to promote the reduction of Fe(III) cations during electrodeposition. This solution was adjusted to pH ~3.0 using 6 M NaOH or 6 M HCl and transferred to a cylindrical plating cell. A platinum wire anode was positioned approximately 1 cm above the silver disc substrate, and a potential of 7.0 ± 0.1 V was applied corresponding to an initial current of 0.09 ± 0.01 A (115 ± 13 mA/cm^2^). Electrical current and pH were measured at multiple time-points during electrodeposition. 20 µL aliquots of the plating solution were also collected at multiple time-points for Fe-concentration measurements by microwave plasma atomic emission spectroscopy (MP-AES, Agilent Technologies, Santa Clara, CA). When electrodeposition had completed as determined by the electrolyte becoming colorless (~24 hours), targets were dried and weighed to determine the plated ^54^Fe mass. Target thicknesses were not measured directly, but rather calculated as the mass of electroplated ^54^Fe divided by the circular plating area (0.79 cm^2^).

Targets were irradiated by 16 MeV protons (PETtrace 800, GE Healthcare, Chicago, IL) with water-jet cooling on the rear target face. Beam currents of up to 60 µA were applied without changes in target appearance. Following irradiation the short-lived ^54^Co (t_1/2_: 1.5 min) impurity was allowed to decay for 10 minutes before dismounting the target. Radioactivities were quantified by efficiency-calibrated high-purity germanium (HPGe) gamma spectroscopy, and end of bombardment (EoB) decay correction was performed using the nominal ^51^Mn half-life (46.2 ± 0.1 min, 95% confidence interval)^23^.

### Mn(II)/Fe(III) Separation Chemistry

Following irradiation, targets were placed in a cylindrical dissolution cell, whereby an o-ring sealed against the front of the target face around the electrodeposited and irradiated ^54^Fe material. After the addition of 2 mL of 11 M HCl, the reaction vessel was brought to 80 °C. Dissolution was found to be complete in less than 20 minutes. To this solution 1.8 mL H_2_O + 0.2 mL 30% H_2_O_2_ was added (see Supplemental Note for Mn/Fe redox details) before transferring to a 15 mL (1.5 cm diameter) AG-1 × 8 strongly-basic anion exchange column which had been equilibrated with ~5 column volumes of 5 M HCl. Using 5 M HCl as mobile phase, the first 5 mL of eluent were discarded. The following 10 mL, containing the ^51^Mn product, were collected in a pear-shaped rotary evaporator flask. The ^51^Mn product was taken to dryness under reduced atmosphere, and the resulting ^51^MnCl_2_ residue was redissolved in ~500 µl of pH 6.5 0.01 M NaOAc buffer. The enriched ^54^Fe target material was recovered from the separation column in 30–50 mL of 0.1 M HCl, which was subsequently taken to dryness (ferric chloride) by boiling under N_2_ gas flow.

The Mn(II) oxidation state following separation was confirmed by thin-layer chromatographic techniques, as previously described^12^. Residual Fe impurities in the final ^51^Mn product were quantified by MP-AES analysis. An effective specific activity was measured by competitive DOTA chelation (room temperature, 0.15 M NaOAc, pH ~6.0, 1 hour) followed by silica thin-layer chromatography (0.25 M NH_4_OH). The mass of DOTA required to bind 50% of a sample’s activity was interpolated from the resulting sigmoidal binding curve, and effective specific activity was calculated as the amount of activity divided by twice this mass.

### Animal Model, PET/CT Imaging

All animal studies were conducted in accordance with relevant guidelines. All animal studies were conducted under a protocol approved by the University of Wisconsin Institutional Animal Care and Use Committee. Non-fasted healthy ICR mice (Envigo, Indianapolis, IN) were divided into two groups. Mice in the first group (n = 2) were anaesthetized by isoflurane (4% induction, 1% maintenance), tail-vein catheters were affixed, and mice were placed on the microPET/CT bed in a prone position (Inveon, Siemens Preclinical Solutions, Knoxville, TN). Dynamic PET acquisition was started and ^51^Mn^2+^ was administered in a rapid bolus (3.3 MBq, 200 µl, 10% 0.01 M NaOAc/90% PBS) through the tail-vein catheter. 60 minutes of dynamic PET data were acquired following ^51^Mn^2+^ administration. Due to the impact of volatile anesthetics on voltage-dependent calcium channel (VDCC) activation^13, 24^, the second group (n = 3) received an intravenous (I.V.) bolus of ^51^Mn^2+^ (1.6 MBq, 200 µl, 10% 0.01 M NaOAc/90% PBS) while awake. 60 minutes post-injection mice were anaesthetized by isoflurane and a 10 minute static PET scan acquired. Injected activities for static PET scans (1.6 MBq) were selected to provide approximately 40 million coincident detection events during this 10 minute PET scan. Injected activities for dynamic PET scans (3.3 MBq) were increased relative to static PET scans to provide sufficient counting statistics during short-duration PET frames. Following imaging, mice were immediately sacrificed by CO_2_ asphyxiation, and organs were extracted. *Ex vivo* biodistribution measurements were performed by gamma counting (Wizard 2480, PerkinElmer, Waltham, MA).

Dynamic PET data were binned into 46 frames (12 × 5 s, 6 × 10 s, 6 × 30 s, 6 × 150 s, 6 × 300 s) and frames were reconstructed using non-scatter-corrected 3D ordered-subset expectation maximization followed by maximum *a posteriori* reconstruction (OSEM3D/MAP). Static PET data were reconstructed into a single frame by OSEM3D/MAP.

### Dosimetry Calculations

Due to the rapid blood clearance of Mn^2+^, OLINDA (Organ Level INternal Dose Assessment) dosimetry calculations^25^ were performed assuming instant compartment localization with organ activity fractions equal to those measured by *ex vivo* biodistribution herein. Based on the previously measured lengthy organ residence times of Mn^2+^, it was also assumed that the effective organ clearance half-life (T_eff_) was equal to the radioactive half-life of ^51^Mn (t_1/2_: 46.2 min)^12^. It was also assumed that ^51^Mn injections were 100% radionuclidically pure. In regards to the daughter isotope, ^51^Cr (t_1/2_: 27.7 d), it was assumed that the activity remained in same organ compartments as the parent ^51^Mn biodistribution without biological clearance. Standard radiation weighting factors were used (γ = 1, β = 1). Source-organ integrated decays for ^51^Mn and ^51^Cr are tabulated in Table S1. Based on these assumptions, effective dose (ED) and effective dose equivalent (EDE) (units of mSv/MBq) were calculated for a standard adult male and female.

## Results

### ^54^Fe Target Fabrication and Irradiation Results

**Figure 1:**
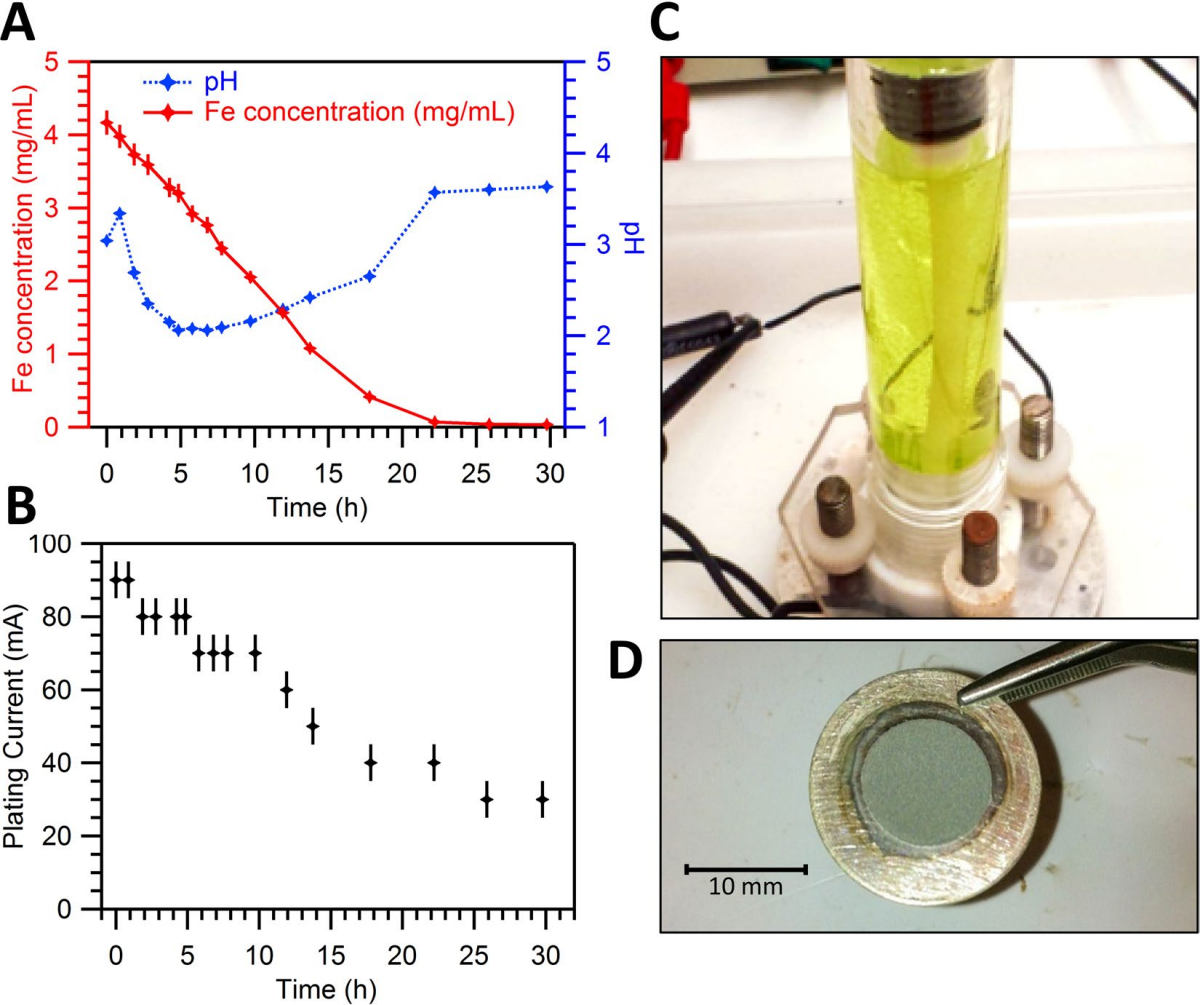
(**A**) Concentration of Fe in electrodeposition solution as a function of time (red) and solution pH as a function of time (blue). Fe concentration was measured by microwave plasma atomic emission spectroscopy (MP-AES). (**B**) Plating current as a function of time with plating potential held constant at 7.0 ± 0.1 V. (**C**) Photograph of plating cell at the start of plating. During plating the light green color becomes colorless. (**D**) Photograph of electroplated ^54^Fe target on Ag disc substrate.

Electrodeposition was found to be complete in approximately 24 hours with residual Fe concentration dropping to <0.04 mg/mL (~0.5 mg ^54^Fe unplated). Changes in plating metrics during electrodeposition are shown in Fig. 1. The electroplated ^54^Fe material appeared dark grey in color, rough in texture, and strongly adhered to the Ag substrate. Occasionally slight oxidation could be seen near the periphery of the electroplated area, but this appeared to reduce during target irradiation. Precipitation was observed during pH adjustment in solutions containing greater than ~100 mg of Fe. This may indicate that larger electrolyte volumes are needed to produce high-mass targets. Targets were irradiated with up to 60 µA of 16 MeV protons, and no change in target appearance was observed. Targets of thicknesses 46.2–64.4 mg/cm^2^ were irradiated by 30 µA of 16 MeV protons for one hour had end of bombardment (EoB) yields of 1.21–1.66 GBq, as measured by efficiency-calibrated HPGe gamma spectroscopy.

### ^51^Mn Separation and ^54^Fe Recovery

Total chemistry duration including dissolution, separation, dry-down, and final formulation was found to be approximately 90 minutes. Decay-corrected ^51^Mn yield was 75.9 ± 3.6% (n = 6). Recovery yields could be improved by collecting more than 10 mL of eluent at the expense of increased separation and dry-down duration. For targets of thicknesses 46–64 mg/cm^2^ (n = 4) irradiated by 30 µA of 16 MeV protons for one hour, end of chemistry (EoC) yield was found to be 190–370 MBq (n = 4).

Final Fe impurity masses for three representative complete production runs are listed in Table 1, along with corresponding separation factors. ^54^Fe recovery efficiency between productions was found to be 93.7 ± 3.5% (n = 7). The final ^51^Mn product, decay-corrected to EoB, was found to be >99.9% radionuclidically pure by HPGe gamma spectroscopy with the ^51^Cr daughter being the largest impurity (0.08%). Trace radionuclidic impurities are listed in Table 2. An EoB effective specific activity of 7.4 GBq/µmol (1.9 GBq/µmol at EoC, n = 1) was measured by titration with DOTA.

**Table 1:** ^51^Mn irradiation yields and separation results from three representative production runs.

Run #	Target Thickness (mg/cm^2^)	EoB Yield (GBq)	Final Fe Impurity Mass (µg)	Separation factor
**1**	64.4	1.66 ± 0.08	8.89 ± 0.08	(3.92 ± 0.03) x10^3^
**2**	58.1	1.31 ± 0.07	0.72 ± 0.01	(3.42 ± 0.05) x10^4^
**3**	46.2	1.21 ± 0.06	0.043 ± 0.001	(6.67 ± 0.15) x10^5^
**4**	61.5	1.58 ± 0.08	4.82 ± 0.10	(7.27 ± 0.29) x10^3^

**Table 2:** Radionuclidic purity of separated ^51^Mn product measured by HPGe gamma spectroscopy.

Isotope	t_1/2_	EoB Activity Fraction	EoC Activity Fraction
^51^Mn	46.2 m	99.91%	99.34%
^52^Mn	5.59 d	0.0001%	0.0004%
^51^Cr	27.7 d	0.08%	0.61%
^55^Co	17.5 h	0.012%	0.045%
^56^Co	77.2 d	0.0009%	0.003%
^57^Co	272 d	0.00002%	0.00009%

### PET results

**Figure 2:**
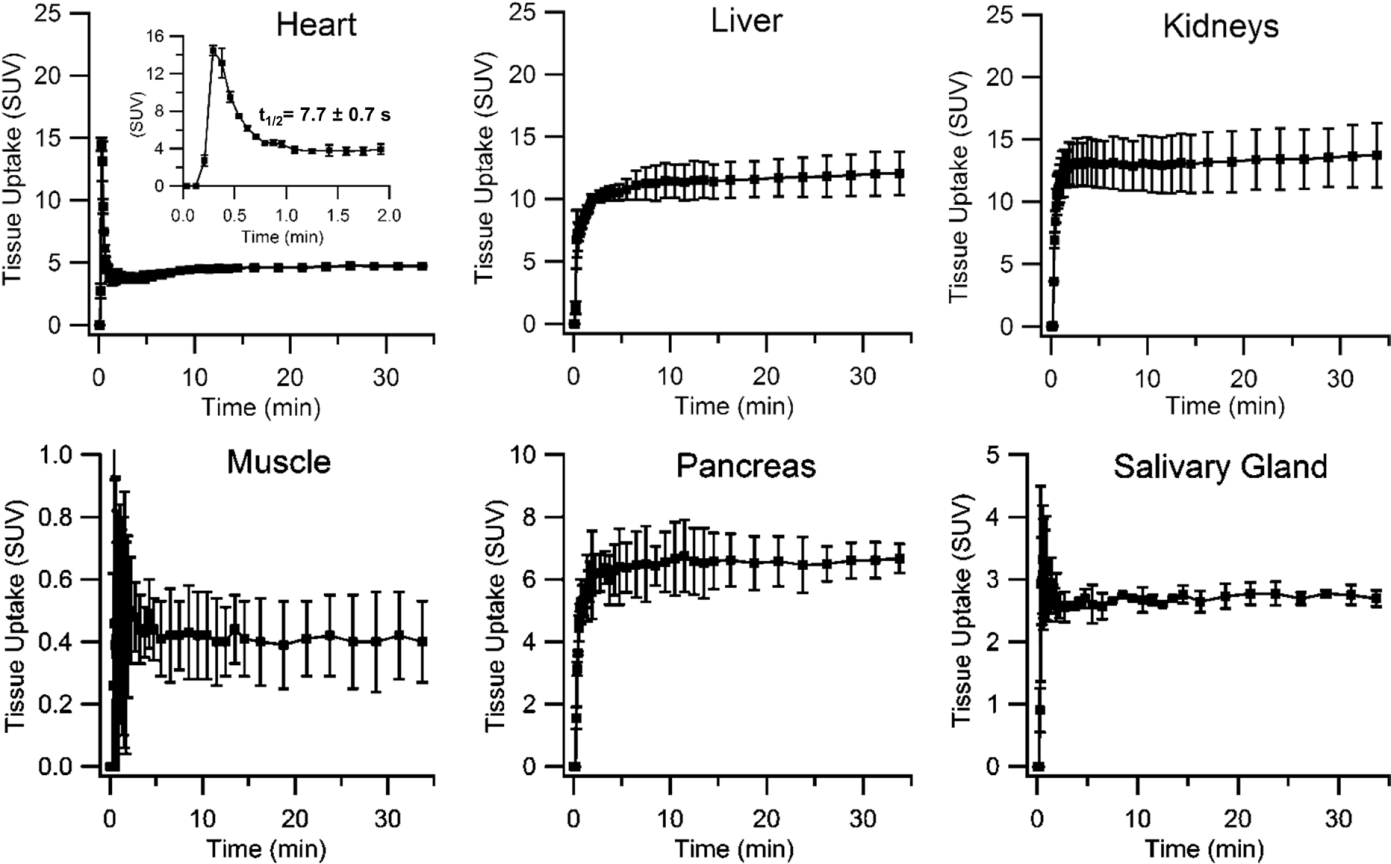
Dynamic PET time-activity curves (TACs) of organ ROIs in ICR mice (n = 2, mean ± SD) injected with a rapid intravenous bolus of ^51^Mn(II), imaged for 30 minutes post-injection.

Rapid ^51^Mn accumulation in the heart, liver, kidneys, pancreas, and salivary glands were observed in ICR mice (n = 5) following a rapid intravenous bolus injection. PET time-activity curves (TACs) are shown in Fig. 2, and tabulated data is listed in Table S4. Following initial distribution (<1 min), uptake was observed to be stable over 30 minutes of PET imaging, which is consistent with previous findings^9, 26, 27^ employing ^52g^Mn^2+^. A heart blood-pool clearance half-life of 7.7 ± 0.7 seconds was determined by weighted exponential least squares regression of the heart TAC from 0.375 to 3.25 minutes post-injection.

**Figure 3:**
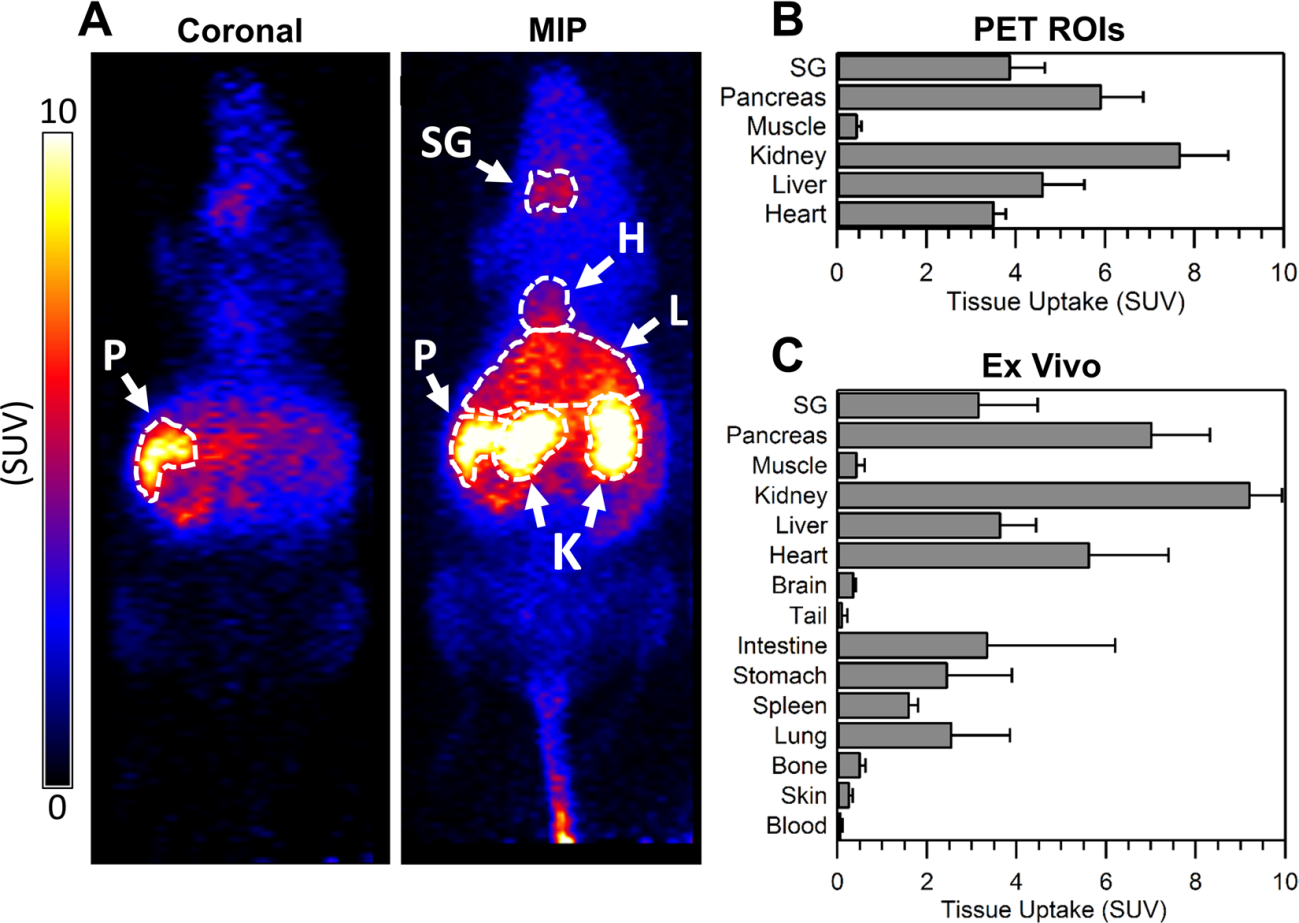
(**A**) Coronal slice and maximum intensity projection (MIP) static PET images of a representative ICR mouse injected intravenously with ^51^Mn(II) (non-anaesthetized during injection). PET images were acquired one hour post-injection. Pancreas (P), salivary gland (SG), heart (H), liver (L) and kidneys (K) indicated by arrows. (**B**) ^51^Mn tissue uptake quantification of hand-drawn PET ROIs in ICR mice (n = 3, mean ± SD) injected with a rapid intravenous bolus of ^51^Mn(II). (**C)** *Ex vivo* ^51^Mn biodistribution in ICR mice (n = 3, mean ± SD) immediately following PET imaging, measured by gamma counting.

Delineation of the pancreas from the surrounding organs (e.g. kidneys) was readily achieved in static PET images (Fig. 3A). PET ROI quantification (Fig. 3B) and *ex vivo* biodistribution by gamma counting (Fig. 3C) show little activity in the muscle and blood, which is consistent with results from dynamic PET imaging. Tabulated PET and *ex vivo* biodistribution data are listed in Table S3 and Table S4. *Ex vivo* biodistribution shows highest ^51^Mn uptake in the kidneys (9.2 ± 0.7 SUV), followed by the pancreas (7.0 ± 1.3 SUV) and the heart (5.6 ± 1.8 SUV). Comparing dynamic PET subjects (n = 2, I.V. ^51^MnCl_2_ bolus under isoflurane) with static PET subjects (n = 3, I.V. ^51^MnCl_2_ non-anaesthetized) reveals significantly higher kidney uptake in anaesthetized dynamic PET subjects, 13.7 ± 2.6 vs. 7.7 ± 1.1 (p = 0.03).

Good agreement was observed between *in vivo* PET quantification and *ex vivo* gamma counting in all tissues with the exception of the heart. As tissues are rinsed and wicked dry prior to weighing and gamma counting, this discrepancy in measured heart uptake is likely due to the inclusion of low-activity blood mass in heart PET ROIs. Intersubject biodistribution variability was found to be minimal when using the SUV uptake metric despite highly varied subject weights (37.6, 48.3, and 22.1 g). As expected, greater intersubject biodistribution variability was observed when using the %ID/g uptake metric.

### Dosimetry Calculation Results

^51^Mn was found to have an EDE of 0.0362 mSv/MBq and 0.0422 mSv/MBq for the standard male and female human model respectively. The daughter isotope ^51^Cr was found to have an EDE of 0.267 mSv/MBq and 0.324 mSv/MBq for the standard male and female model respectively. OLINDA dosimetry predictions for a typical clinical dose of ^51^Mn (370 MBq, 10 mCi) are listed in Table 3.

**Table 3:** Effective dose equivalent (EDE) for a 370 MBq intravenous injection of radionuclidically pure ^51^Mn^2+^ in the standard adult human male and female.

Contribution	Male (mSv)	Female (mSv)
^51^Mn	13.4	15.6
^51^Cr	0.11	0.14
Total	13.5	15.8

## Discussion

Manganese is an essential trace element in mammalian biology^28^ and has many prospective applications as an imaging agent in medicine. Of the three positron-emitting isotopes of manganese, ^51^Mn is best suited to clinical PET based on decay characteristics. Robust methods for the preparation of ^51^Mn are essential to the investigation of basic science and clinical questions relating to the biological role of manganese in disease.

To our knowledge, this work constitutes the first attempt at ^51^Mn production via ^54^Fe(p,α) and radiochemical isolation in clinically-relevant quantities. The electrodeposition method described here has proved effective for the quantitative reduction of ^54^Fe(III) to ^54^Fe metal, with the electroplated Fe metal being strongly adhered to the Ag disc substrate. From Fig. 1A, it may be inferred that ^54^Fe(III) reduction follows zero-order kinetics for the majority of the plating duration. This suggests that plating time may vary depending on the ^54^Fe mass in solution. The plating solution pH was found to be highly variable during electrodeposition, with the solution rising above pH 3.0 upon completion. This acute rise in pH near plating completion may enable non-colorimetric automation methods.

The fabricated ^54^Fe targets were robust, withstanding relatively high beam currents (16 MeV, 60 µA) without changes in appearance. The target thicknesses (45–65 mg/cm^2^) and irradiation parameters (30 µA for 1 h) used in this work were sufficient to provide enough EoC activity (190–370 MBq) for several small animal studies or approximately one human study. EoC yield could readily be increased to 1.5–2.0 GBq by employing target thicknesses of approximately 100 mg/cm^2^ and irradiating with a beam current of 60 µA for 2 hours. Based on these yields, a chemistry duration of ~90 minutes is sufficiently short for production purposes. However at institutions without solid-target capabilities, a solution-target of ^54^Fe(NO_3_)_2_ or ^50^Cr(NO_3_)_3_ could provide elegant alternative production routes. Although the chemical isolation of ^51^Mn from bulk Fe metal is simpler than ^51^Mn from bulk Cr, the production cross section for ^50^Cr(d,n) is significantly higher than ^54^Fe(p,α) which may help compensate for the reduced target atomic fraction in solution targets^17, 29, 30^.

PET imaging of pancreatic beta cells with ^51^MnCl_2_ appears promising due to the rapid blood clearance and significant pancreatic accumulation. Further studies are needed to determine the feasibility and optimal study methodology for beta cell mass quantification by ^51^Mn-PET. To this end, non-specific exocrine uptake could possibly be quantified by co-injection of VDCC blocking agents such as nifedipine. Furthermore, serial studies are warranted for monitoring the decline in beta cell mass in a streptozotocin-induced mouse model of type-I diabetes^31, 32^. Other positron-emitting divalent metals such as ^63^Zn^2+^ (t_1/2_: 38.5 min β^+^: 92.7%, Eβ_ave_: 0.92 MeV) may also prove useful for beta cell related investigations, as VDCCs are permeable to Zn^2+^ and significant ^63^Zn pancreatic uptake has been observed in mice by Degrado *et al*.^33, 34^.

The heart blood-pool clearance half-life of ^51^Mn^2+^ found in this work (7.7 ± 0.7 s) is astonishingly rapid, suggesting first-pass tissue localization kinetics. It is likely that this measured clearance half-life significantly differs from a true blood clearance half-life, as the assumption of uniformly-distributed tracer within the blood pool is likely inaccurate with such rapid clearance kinetics. Rather, it is likely that we are simply observing the bolus passage through the heart volume following injection. Rapid blood clearance and stable accumulation offers experimental flexibility with regards to PET imaging duration and timing following tracer administration. Tracer kinetics such as these also support the use of the SUV uptake metric for ^51^Mn-PET studies, as tracers without significant tissue clearance, i.e. [^18^F]-FDG, lend themselves well to such analytic methods. Furthermore, the rapid blood clearance of ^51^Mn^2+^ may enable multiple-injection protocols within a single patient study. Techniques such as these may prove useful in beta cell mass (BCM) quantification studies for the subtraction of non-specific exocrine pancreas uptake by stimulation or blocking (i.e. glibenclamide or nifedipine) of beta cell VDCCs following baseline imaging. On the other hand, the pulsatile nature of calcium transport^35, 36^ may increase test-retest variability for bolus injection techniques. This effect could possibly be mitigated by administering ^51^MnCl_2_ as an intravenous infusion over 5–15 minutes.

The mean positron energy emitted during the decay of ^51^Mn (962 keV) is significantly higher than that of ^18^F (250 keV) or ^52g^Mn (242 keV) which leads to poorer spatial resolution in PET images. Regardless the resolution of ^51^Mn has still proven to be sufficient for whole-organ-ROI microPET studies, and positron range is not typically the limiting factor of clinical PET resolution^37^.

^51^Mn dosimetry appears favorable, even when accounting for the long-lived daughter ^51^Cr, and making the conservative assumption that this daughter is not biologically excreted. In this work, a cumulative effective dose equivalent of ~15 mSv for a 370 MBq ^51^Mn PET study was calculated. This result is comparable to the average dose for an [^18^F]-FDG study of 14.1 mSv^38^. This suggests that it would be possible to perform up to three repeat PET studies in healthy or type-I diabetic volunteers without exceeding the annual non-stochastic International Commission on Radiological Protection (ICRP) limit of 50 mSv for research subjects^39, 40^.

## Conclusion

Methods for the efficient production and isolation of ^51^Mn by ^54^Fe(p,α) followed by anion exchange chromatography have been described. Initial ^51^MnCl_2_ pharmacokinetic characterization in mice and predicted human dosimetry show promise for a variety of PET applications, including VDCC activation imaging in pancreatic beta cells.

## Electronic supplementary material

Supplementary Information
