# Supplementary material for: Preparation and in vivo characterization of 51MnCl2 as PET tracer of Ca2+ channel-mediated transport
Source: Sci Rep. 2017 Jun 8;7:3033. doi: 10.1038/s41598-017-03202-0 (PMC5465055; doi:10.1038/s41598-017-03202-0)
Supplement: Supplementary file 1 — Supplementary Information [file 41598_2017_3202_MOESM1_ESM.pdf]

Supplementary Information

Preparation and in vivo characterization of $^{51}MnCl_2$ as PET tracer of Ca^{2+} channel-mediated transport

Stephen A. Graves[1], Reinier Hernandez[1], Hector F. Valdovinos[1], Paul A. Ellison[1], Jonathan W. Engle[1], Todd E. Barnhart[1], Weibo Cai[1,2,3], Robert J. Nickles[1]*

[1]Department of Medical Physics, University of Wisconsin – Madison
[2]Carbone Cancer Center, University of Wisconsin – Madison
[3]Department of Radiology, University of Wisconsin – Madison

*Corresponding author information:
Robert J. Nickles
rnickles@wisc.edu
1005 WIMR, 1111 Highland Ave., Madison, WI, 53705, USA
Phone: +1 (608) 263-5805

Jonathan W. Engle
jwengle@wisc.edu
1005 WIMR, 1111 Highland Ave., Madison, WI, 53705, USA
Phone: +1 (608) 263-7932

Supplementary Note: Fe/Mn Redox Chemistry

To determine whether Mn^{2+} and Fe^{2+} are oxidized to Mn^{3+} and Fe^{3+} under acidic conditions by the addition of H_2O_2 prior to anion exchange chromatography, consider the following reactions and associated standard reduction potentials:

$$Mn^{3+}(aq) + e^{-} \longrightarrow Mn^{2+}(aq) \qquad E^{\circ} = +1.51\,V$$

$$Fe^{3+}(aq) + e^{-} \longrightarrow Fe^{2+}(aq) \qquad E^{\circ} = +0.77\,V$$

$$H_2O_2(aq) + 2H^{+} + 2e^{-} \longrightarrow 2H_2O \qquad E^{\circ} = +1.78\,V$$

For the oxidation of Mn^{2+} by H_2O_2, we obtain:

$$2Mn^{2+}(aq) + H_2O_2(aq) + 2H^{+} + 2e^{-} \longrightarrow 2H_2O + 2Mn^{3+} + 2e^{-} \qquad E^{\circ} = 1.78 - 2(1.51)\ V$$

$$\boxed{2Mn^{2+} + H_2O_2(aq) + 2H^{+} \longrightarrow 2H_2O + 2Mn^{3+} \qquad E^{\circ} = -1.24\,V}$$

For the oxidation of Fe^{2+} by H_2O_2, we obtain:

$$2Fe^{2+}(aq) + H_2O_2(aq) + 2H^{+} + 2e^{-} \longrightarrow 2H_2O + 2Fe^{3+} + 2e^{-} \qquad E^{\circ} = 1.78 - 2(0.77)\ V$$

$$\boxed{2Mn^{2+} + H_2O_2(aq) + 2H^{+} \longrightarrow 2H_2O + 2Mn^{3+} \qquad E^{\circ} = +0.24\,V}$$

From these results, we can see that Mn^{2+} is not oxidized to Mn^{3+}, whereas the oxidation of Fe^{2+} to Fe^{3+} is spontaneous. The spontaneous oxidation of Fe^{2+} to Fe^{3+} is evidenced experimentally by a color change, from pale yellow to deep orange/brown.

Tissue	Number of ^{51}Mn disintigrations (MBq-h/MBq)	Number of ^{51}Cr disintigrations (MBq-h/MBq)
Adrenals	0.00E+00	0.00E+00
Brain	4.43E-03	3.93E+00
Breasts	0.00E+00	0.00E+00
Gallbladder Contents	0.00E+00	0.00E+00
LLI	0.00E+00	0.00E+00
Small Intestine	0.00E+00	0.00E+00
Stomach	0.00E+00	1.23E+01
ULI	6.42E-03	5.70E+00
Heart Contents	0.00E+00	0.00E+00
Heart Wall	2.37E-02	2.10E+01
Kidneys	6.91E-02	6.12E+01
Liver	6.30E-02	5.58E+01
Lungs	1.68E-02	1.49E+01
Muscle	2.18E-03	1.93E+00
Ovaries	0.00E+00	0.00E+00
Pancreas	4.11E-02	3.64E+01
Red Marrow	0.00E+00	0.00E+00
Cortical Bone	8.40E-04	7.48E-01
Trabecular Bone	0.00E+00	0.00E+00
Spleen	9.16E-03	8.12E+00
Testes	0.00E+00	0.00E+00
Thymus	0.00E+00	0.00E+00
Thyroid	0.00E+00	0.00E+00
Urinary Bladder Contents	0.00E+00	0.00E+00
Uterus/Uterine Wall	0.00E+00	0.00E+00
Total Body	8.70E-01	7.20E+02

Table S1. Source organ integrated disintigrations for ^{51}Mn and ^{51}Cr used in OLINDA dose calculations.

Time (min)	Heart (SUV)	Liver (SUV)	Kidneys (SUV)	Muscle (SUV)	Pancreas (SUV)	Salivary gland (SUV)
0.04	0.00 ± 0.00	0.00 ± 0.00	0.00 ± 0.00	0.00 ± 0.00	0.00 ± 0.00	0.00 ± 0.00
0.13	0.00 ± 0.00	0.00 ± 0.00	0.00 ± 0.00	0.00 ± 0.00	0.00 ± 0.00	0.00 ± 0.00
0.21	2.73 ± 0.58	1.32 ± 0.50	0.04 ± 0.05	0.00 ± 0.00	0.00 ± 0.00	0.00 ± 0.00
0.29	14.48 ± 0.53	6.73 ± 2.32	3.62 ± 0.03	0.00 ± 0.00	1.56 ± 0.35	0.90 ± 0.35
0.38	13.12 ± 1.58	7.21 ± 1.90	6.91 ± 0.63	0.26 ± 0.36	3.13 ± 0.22	2.93 ± 1.57
0.46	9.48 ± 0.58	6.99 ± 1.17	8.41 ± 0.85	0.46 ± 0.63	3.63 ± 0.07	2.97 ± 0.70
0.54	7.47 ± 0.06	7.33 ± 0.67	9.69 ± 1.36	0.39 ± 0.53	4.43 ± 0.42	3.32 ± 0.87
0.63	6.16 ± 0.24	7.49 ± 0.51	10.53 ± 1.61	0.41 ± 0.52	4.71 ± 0.03	3.08 ± 0.89
0.71	5.27 ± 0.20	7.50 ± 0.30	10.75 ± 1.30	0.36 ± 0.46	5.08 ± 0.12	2.83 ± 0.45
0.79	4.59 ± 0.19	7.97 ± 0.38	11.02 ± 1.27	0.32 ± 0.40	5.28 ± 0.72	3.14 ± 0.65
0.88	4.63 ± 0.24	7.98 ± 0.34	11.18 ± 1.26	0.26 ± 0.31	5.06 ± 0.09	3.18 ± 0.83
0.96	4.46 ± 0.31	8.33 ± 0.11	11.65 ± 1.32	0.20 ± 0.25	5.31 ± 0.50	3.16 ± 0.14
1.08	3.88 ± 0.41	8.34 ± 0.16	11.94 ± 1.55	0.49 ± 0.35	5.14 ± 0.32	2.57 ± 0.24
1.25	3.72 ± 0.25	8.70 ± 0.13	12.21 ± 1.83	0.45 ± 0.31	5.53 ± 0.43	2.81 ± 0.14
1.42	3.79 ± 0.60	9.27 ± 0.24	12.72 ± 1.45	0.45 ± 0.35	5.72 ± 1.06	2.94 ± 0.41
1.58	3.71 ± 0.46	9.10 ± 0.17	12.57 ± 1.42	0.47 ± 0.41	5.78 ± 0.50	2.73 ± 0.18
1.75	3.73 ± 0.45	9.57 ± 0.07	12.67 ± 1.51	0.38 ± 0.34	6.02 ± 0.54	2.66 ± 0.20
1.92	3.92 ± 0.55	10.06 ± 0.32	13.06 ± 1.68	0.48 ± 0.26	6.14 ± 1.41	2.71 ± 0.39
2.25	3.81 ± 0.41	10.01 ± 0.29	12.89 ± 1.73	0.50 ± 0.17	6.19 ± 0.61	2.58 ± 0.20
2.75	3.78 ± 0.41	10.23 ± 0.46	13.16 ± 1.93	0.48 ± 0.11	6.20 ± 0.59	2.56 ± 0.24
3.25	3.79 ± 0.36	10.37 ± 0.49	13.01 ± 1.68	0.44 ± 0.11	6.38 ± 0.53	2.58 ± 0.03
3.75	3.65 ± 0.29	10.46 ± 0.58	13.20 ± 1.95	0.43 ± 0.08	5.98 ± 0.79	2.58 ± 0.06
4.25	3.80 ± 0.33	10.51 ± 0.61	13.15 ± 1.88	0.49 ± 0.11	6.30 ± 0.81	2.65 ± 0.04
4.75	3.83 ± 0.44	10.53 ± 0.66	13.02 ± 1.88	0.44 ± 0.10	6.40 ± 1.22	2.69 ± 0.15
5.50	3.96 ± 0.38	10.77 ± 0.84	12.97 ± 1.90	0.41 ± 0.12	6.38 ± 0.79	2.60 ± 0.31
6.50	4.10 ± 0.31	11.11 ± 1.14	13.13 ± 2.08	0.42 ± 0.15	6.48 ± 1.05	2.57 ± 0.21
7.50	4.20 ± 0.12	11.24 ± 1.30	12.95 ± 2.09	0.42 ± 0.11	6.49 ± 1.21	2.65 ± 0.02
8.50	4.37 ± 0.10	11.26 ± 1.41	12.85 ± 1.98	0.43 ± 0.15	6.43 ± 0.63	2.75 ± 0.01
9.50	4.43 ± 0.09	11.47 ± 1.40	13.09 ± 2.21	0.42 ± 0.14	6.56 ± 0.96	2.69 ± 0.03
10.50	4.51 ± 0.03	11.44 ± 1.32	12.97 ± 2.25	0.42 ± 0.14	6.65 ± 1.18	2.67 ± 0.20
11.50	4.49 ± 0.07	11.35 ± 1.44	12.92 ± 2.21	0.40 ± 0.14	6.75 ± 1.16	2.67 ± 0.08
12.50	4.53 ± 0.03	11.50 ± 1.55	13.00 ± 2.32	0.40 ± 0.11	6.58 ± 1.06	2.60 ± 0.02
13.50	4.50 ± 0.05	11.51 ± 1.43	13.13 ± 2.30	0.44 ± 0.11	6.52 ± 1.12	2.69 ± 0.03
14.50	4.56 ± 0.05	11.41 ± 1.38	13.02 ± 2.31	0.41 ± 0.12	6.59 ± 0.90	2.76 ± 0.14
16.25	4.59 ± 0.07	11.53 ± 1.46	13.17 ± 2.39	0.40 ± 0.14	6.62 ± 0.84	2.64 ± 0.17
18.75	4.59 ± 0.01	11.59 ± 1.45	13.20 ± 2.47	0.39 ± 0.14	6.52 ± 0.86	2.73 ± 0.20
21.25	4.61 ± 0.07	11.71 ± 1.51	13.38 ± 2.43	0.41 ± 0.12	6.58 ± 0.80	2.77 ± 0.18
23.75	4.68 ± 0.02	11.74 ± 1.57	13.42 ± 2.49	0.42 ± 0.13	6.46 ± 0.89	2.77 ± 0.19
26.25	4.73 ± 0.01	11.81 ± 1.65	13.40 ± 2.38	0.40 ± 0.15	6.49 ± 0.56	2.69 ± 0.10
28.75	4.72 ± 0.06	11.89 ± 1.64	13.55 ± 2.33	0.40 ± 0.16	6.60 ± 0.58	2.77 ± 0.05
31.25	4.72 ± 0.02	12.02 ± 1.75	13.65 ± 2.50	0.42 ± 0.14	6.62 ± 0.58	2.75 ± 0.16
33.75	4.72 ± 0.02	12.06 ± 1.73	13.74 ± 2.59	0.40 ± 0.13	6.67 ± 0.47	2.69 ± 0.13

Table S2. Tabulated time activity curves (TACs) measured by dynamic PET for isoflurane anaesthetized ICR mice (n=2) injected with a rapid intravenous bolus of $^{51}MnCl_2$.ROIs were hand-drawn on composite images and applied to all frames. Values represent mean ± SD. A heart blood clearance half-life of 7.7 ± 0.7 s was measured by weighted exponential least-squares regression.

Tissue	Uptake (SUV)
Heart	3.53 ± 0.25
Liver	4.63 ± 0.91
Kidney	7.70 ± 1.06
Muscle	0.47 ± 0.07
Pancreas	5.93 ± 0.93
Salivary Gland	3.90 ± 0.75

Table S3. ^{51}Mn uptake in non-anaesthetized ICR mice (n=3) 1 hour following a rapid intravenous bolus of $^{51}MnCl_2$, quantified by hand-drawn static PET ROIs.

Tissue	Uptake (SUV)
Blood	0.09 ± 0.02
Skin	0.28 ± 0.06
Muscle	0.45 ± 0.16
Bone	0.52 ± 0.11
Heart	5.64 ± 1.75
Lung	2.57 ± 1.28
Liver	3.66 ± 0.78
Kidney	9.23 ± 0.70
Spleen	1.62 ± 0.18
Pancreas	7.03 ± 1.28
Stomach	2.47 ± 1.43
Intestine	3.37 ± 2.84
Tail	0.12 ± 0.10
Brain	0.38 ± 0.03
Salivary Gland	3.18 ± 1.30

Table S4. ^{51}Mn uptake in ICR mice immediately following PET imaging (~90 min post-injection), quantified by *ex vivo* gamma counting.
